# Neuromodulation With Thoracic Dorsal Root Ganglion Stimulation Reduces Ventricular Arrhythmogenicity

**DOI:** 10.3389/fphys.2021.713717

**Published:** 2021-10-07

**Authors:** Yuki Kuwabara, Siamak Salavatian, Kimberly Howard-Quijano, Tomoki Yamaguchi, Eevanna Lundquist, Aman Mahajan

**Affiliations:** ^1^Department of Anesthesiology and Perioperative Medicine, University of Pittsburgh, Pittsburgh, PA, United States; ^2^Department of Anesthesiology and Perioperative Medicine, University of Pittsburgh Medical Center, Pittsburgh, PA, United States

**Keywords:** autonomic nervous system, sympathetic hyperactivity, neuromodulation, ventricular arrhythmias, dorsal root ganglia (DRG)

## Abstract

**Introduction:** Sympathetic hyperactivity is strongly associated with ventricular arrhythmias and sudden cardiac death. Neuromodulation provides therapeutic options for ventricular arrhythmias by modulating cardiospinal reflexes and reducing sympathetic output at the level of the spinal cord. Dorsal root ganglion stimulation (DRGS) is a recent neuromodulatory approach; however, its role in reducing ventricular arrhythmias has not been evaluated. The aim of this study was to determine if DRGS can reduce cardiac sympathoexcitation and the indices for ventricular arrhythmogenicity induced by programmed ventricular extrastimulation. We evaluated the efficacy of thoracic DRGS at both low (20 Hz) and high (1 kHz) stimulation frequencies.

**Methods:** Cardiac sympathoexcitation was induced in Yorkshire pigs (*n* = 8) with ventricular extrastimulation (S1/S2 pacing), before and after DRGS. A DRG-stimulating catheter was placed at the left T2 spinal level, and animals were randomized to receive low-frequency (20 Hz and 0.4 ms) or high-frequency (1 kHz and 0.03 ms) DRGS for 30 min. High-fidelity cardiac electrophysiological recordings were performed with an epicardial electrode array measuring the indices of ventricular arrhythmogenicity—activation recovery intervals (ARIs), electrical restitution curve (S_max_), and Tpeak–Tend interval (Tp-Te interval).

**Results:** Dorsal root ganglion stimulation, at both 20 Hz and 1 kHz, decreased S1/S2 pacing-induced ARI shortening (20 Hz DRGS −21±7 ms, Control −50±9 ms, *P* = 0.007; 1 kHz DRGS −13 ± 2 ms, Control −46 ± 8 ms, *P* = 0.001). DRGS also reduced arrhythmogenicity as measured by a decrease in S_max_ (20 Hz DRGS 0.5 ± 0.07, Control 0.7 ± 0.04, *P* = 0.006; 1 kHz DRGS 0.5 ± 0.04, Control 0.7 ± 0.03, *P* = 0.007), and a decrease in Tp-Te interval/QTc (20 Hz DRGS 2.7 ± 0.13, Control 3.3 ± 0.12, *P* = 0.001; 1 kHz DRGS 2.8 ± 0.08, Control; 3.1 ± 0.03, *P* = 0.007).

**Conclusions:** In a porcine model, we show that thoracic DRGS decreased cardiac sympathoexcitation and indices associated with ventricular arrhythmogenicity during programmed ventricular extrastimulation. In addition, we demonstrate that both low-frequency and high-frequency DRGS can be effective neuromodulatory approaches for reducing cardiac excitability during sympathetic hyperactivity.

## Introduction

Malignant ventricular arrhythmias are the largest cause of sudden cardiac death and are associated with autonomic imbalances (Proietti et al., 2016; Batul et al., 2017). Augmented sympathetic tone leads to ventricular tachycardias (VTs) in several cardiac diseases and is associated with worsening prognosis (Rubart and Zipes, 2005; Zucker, 2006; Shen and Zipes, 2014). Myocardial stress activates the cardio-spinal autonomic neural reflex—beginning with the activation of the neurons that project from the heart to the dorsal root ganglion, which then relay the afferent cardiac signals to the spinal cord where neuronal network interactions between the dorsal horn neurons and intermediolateral nucleus control the efferent sympathetic output (Fukuda et al., 2015; Omura et al., 2021).

Neuromodulation therapies are emerging as novel therapeutic options to modulate the autonomic tone in cardiac patients (Dusi et al., 2019; Herring et al., 2019). For example, we and others have previously shown that spinal cord stimulation targeting the dorsal columns reduced the cardiac sympathoexcitation, decreased ventricular arrhythmias, and improved myocardial function in the porcine model (Southerland et al., 2007; Lopshire et al., 2009; Howard-Quijano et al., 2017a).

Bioelectronic dorsal root ganglion stimulation (DRGS) is a novel approach to neuromodulation therapy, targeting these neural structures that contain the axons and cell bodies of afferent neurons, located at the distal end of the dorsal root of the spinal cord (Sverrisdottir et al., 2020). Early evidence suggests that DRGS is an effective treatment for some pain syndromes, clinically and preclinically (Liem et al., 2015; Deer et al., 2019; Yu et al., 2020).

Spinal cord neural networks are modified by afferent transduction from cardiopulmonary sensory neurites from the DRG neurons (Barman and Yates, 2017; Dale et al., 2020; Omura et al., 2021); therefore, bioelectronic DRGS has the potential to directly modulate the dorsal root and dorsal horn neurons and indirectly modulate the spinal preganglionic neurons that innervate the heart. While DRGS for neuropathic pain has been examined, the effects and roles of DRGS in the treatment of cardiac arrhythmias are not known.

We hypothesized that thoracic DRGS reduces cardiac sympathoexcitation and arrhythmogenicity indices. In this study, we evaluated the effectiveness of DRGS as an alternative neuromodulation therapy by demonstrating the reduction of cardiac sympathoexcitation and ventricular arrhythmogenicity indices induced by programmed ventricular extrastimulation. To investigate our aim, we tested two different stimulation parameters, conventional low-frequency stimulation at 20 Hz and high-frequency stimulation at 1 kHz, targeting the dorsal root ganglion at the T2 spinal level on the left side. The proposed benefit for high-frequency stimulation compared to low frequencies is the avoidance of uncomfortable paresthesia in clinical settings; thus, we investigated DRGS at both frequencies.

## Materials and Methods

The study protocol was approved by the Institutional Animal Care and Use Committee (IACUC) at the University of Pittsburgh. All experiments were performed in compliance with the National Institution of Health *Guide for the Care and Use of Laboratory Animals*.

### Animal Preparation

Eight Yorkshire pigs (four male and four female), weighing 47 ± 4 kgs, were randomized to DRG stimulation protocols. In two pigs, this protocol was performed for both 20 Hz and 1 k Hz, where enough time was allowed to return to the baseline condition before repeating the protocol for another stimulation parameter. We have previously extensively characterized this experimental animal model (Batul et al., 2017; Howard-Quijano et al., 2017b; Dale et al., 2020; Omura et al., 2021). In brief, animals were sedated with telazol (4 mg/kg, intramuscular) and xylazine (2 mg/kg, intramuscular) and mechanically ventilated by tracheal intubation (Omura et al., 2021). General anesthesia was induced and maintained with inhaled isoflurane (2 to 5%) during surgical preparation. Heart rate and cardiac electrocardiogram (ECG) were monitored throughout the experiment using a Prucka CardioLab system (GE Healthcare, United States). The carotid and femoral arteries were catheterized for blood pressure monitoring. In addition, jugular and femoral veins were cannulated for intravenous saline infusion (10 ml/kg) and drug administration. In order to maintain acid–base equilibrium, arterial blood gases were tested hourly, with the adjustment of ventilation or administration of sodium bicarbonate, as necessary. Body temperature was maintained by an external warmer. Animals were placed in the prone position and underwent laminectomy to expose the spinal cord and the left T2 dorsal root. Subsequent to this, a median sternotomy was performed in the supine position to expose the heart. After the completion of surgical preparation, general anesthesia was transitioned to intravenous alpha-chloralose (50 mg/kg initial bolus followed by a 20 mg/kg/h continuous infusion), which limits the impact on cardiac myocardial electrophysiology (Batul et al., 2017; Howard-Quijano et al., 2017b; Dale et al., 2020; Omura et al., 2021). The depth of anesthesia was assessed throughout the experiments by monitoring corneal reflexes, jaw tone, and hemodynamic indices. At the end of the experiment, animals were euthanized by inducing ventricular fibrillation *via* the injection of potassium chloride under deep anesthesia.

### Experimental Protocols

**Figure 1:**
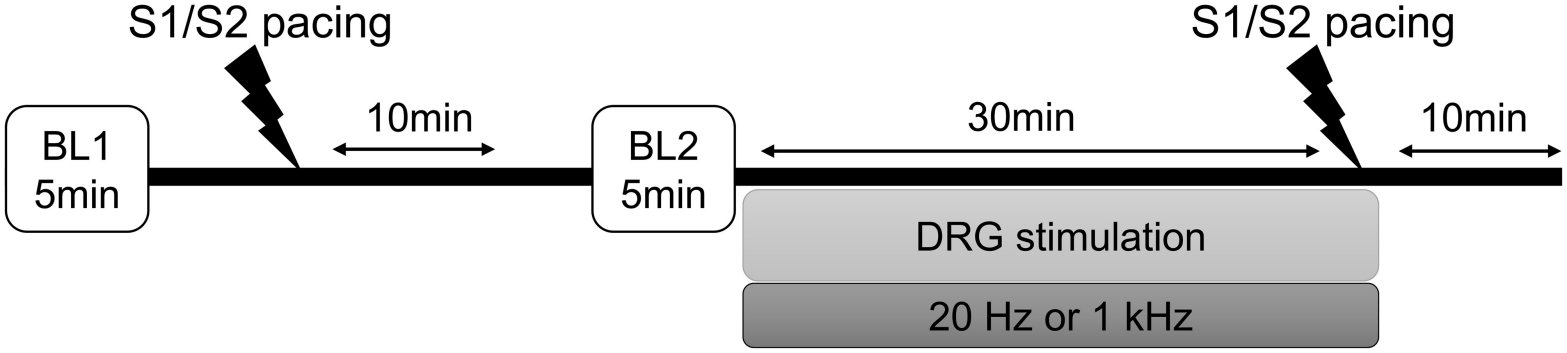
Experimental protocol. Programmed ventricular extrastimulation (S1/S2 ventricular pacing) was performed on eight Yorkshire pigs to induce cardiac sympathoexcitation and testing for ventricular arrhythmogenicity. The efficacy of the dorsal root ganglion stimulation (DRGS) was assessed in reducing sympathoexcitation and cardiac excitability. A 10-min period was allowed after each pacing for return to baseline conditions. In two pigs, this protocol was performed for both 20 Hz and 1 kHz, where enough time was allowed to return to the baseline condition before repeating the protocol for another stimulation parameter. BL, baseline.

The timeline of the experimental protocol is shown in Figure 1. To test the hypothesis that DRGS would reduce cardiac excitability and arrhythmogenicity indices, epicardial cardiac electrophysiological mapping was performed at baseline and in response to the programmed ventricular extrastimulation (experimental protocol), before and after DRGS (Pak et al., 2004).

### Dorsal Root Ganglion Stimulation

While the pig was in the left lateral position, an Axium™ DRG lead (St. Jude Medical, United States) was advanced alongside the dorsal root of the spinal cord and placed next to the left T2 dorsal root ganglion. An A-M systems stimulator (Model 2100, A-M systems, Sequim, WA) and a constant-current isolation unit produced the stimuli for the DRGS. The motor threshold (MT) was assessed by increasing stimulus intensity (current) until muscle contractions were observed in the shoulder, using a stimulus frequency of 2 Hz and a pulse width of 0.4 ms to allow for the reliable assessment of muscle contractions (Howard-Quijano et al., 2017a). Animals were randomized to two different DRGS parameters: a conventional low-frequency stimulation at 20 Hz, 0.4 ms pulse width, or a high-frequency stimulation at 1 k Hz, 0.03 ms pulse width, and a current amplitude of 90% of MT.

### Cardiac Electrophysiology and Ventricular Arrhythmogenicity

#### (a) Activation Recovery Interval Analysis

**Figure 2:**
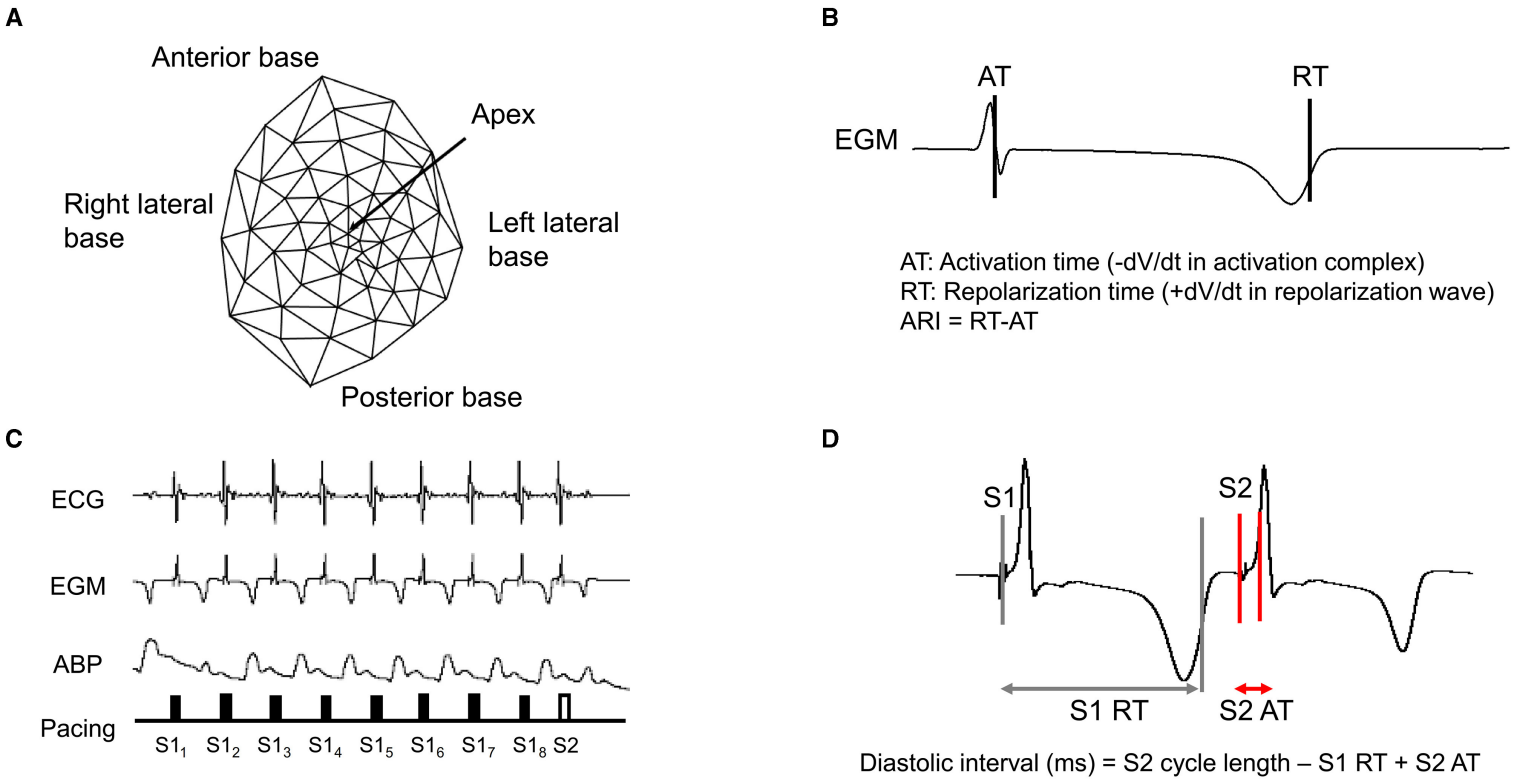
Epicardial electrodes and representative example of S1/S2 pacing. **(A)** A 56-electrode high-fidelity epicardial sock array was placed around the heart to measure ventricular epicardial electrograms, **(B)** Activation recovery interval (ARI), **(C)** Representative example of electrocardiogram (ECG), electrogram (EGM), and arterial blood pressure (ABP) during S1/S2 pacing, **(D)** Diastolic interval. RT, repolarization time; AT, activation time.

A 56-electrode nylon mesh sock electrode was placed around the heart, and unipolar electrograms were measured using a Prucka CardioLab electrophysiology mapping system (GE Healthcare, Fairfield, CT) and unipolar electrograms were recorded (0.05–500 Hz) with the GE CardioLab System (Figures 2A,B) (Vaseghi et al., 2013). We assessed the activation recovery interval (ARI), which is a surrogate of local action potential duration (APD) (Millar et al., 1985; Chinushi et al., 2001). Global ARIs, corrected by heart rate (cARI), were used in this study. ARIs were calculated using customized software (iScalDyn, University of Utah, Salt Lake City, UT) as previously described (Ajijola et al., 2013). All physiological measures were recorded at the following time points: (1) baseline-1, (2) after S1S2 pacing without DRGS, (3) baseline-2, (4) during 30 min of DRGS, and (5) after S1S2 pacing with DRGS (Figure 1). Ten minutes was allowed post-pacing termination for the ventricular arrhythmogenicity and hemodynamic indices to return to baseline. Changes in ARIs were evaluated by comparing the ARI between the baseline or after 30 min DRGS (DRG30) and at 1 min post-ventricular pacing. ARI reduction due to sympathoexcitation caused by ventricular pacing was assessed before and after DRGS.

#### (b) Programmed Ventricular Extrastimulation (S1/S2 Pacing) and Electrical Restitution

Epicardial pacing of the ventricle with programmed extrastimulation (S1 and S2 protocol) was used to create myocardial stress and test ventricular arrhythmogenicity as previously described (Taggart et al., 2003; Howard-Quijano et al., 2017b). The pacing electrodes were placed around the right ventricle outflow tract (RVOT), and the myocardium was placed using a Micropace EPS 320 Cardiac Stimulator System (Micropace, Tustin, CA, United States). An application of a train (S1), which consists of eight paced beats at a cycle length equivalent to 70–75% of the baseline cycle length, was initiated, followed by a premature extrastimulus (S2) generated at 60% of the baseline cycle length and progressively shortened by 10 ms until the effective refractory period was reached (Figures 2C,D).

Electrical restitution curves describing the relationship between ARI and prior diastolic interval were formulated. The maximum slope of the electrical restitution curve is a measure of myocardial arrhythmogenic potential, with greater slopes indicating a higher risk of ventricular arrhythmogenicity (Taggart et al., 2003; Osadchii, 2012, 2019). ARI and diastolic interval were measured, and electrical restitution curves were composed, and the maximum steep slope (S_max_) was calculated using a logarithmic approximation approach (Howard-Quijano et al., 2017b). The maximum slope of restitution was measured by analyzing the first derivative of the fitted curve (Ng et al., 2007).

#### (c) Tpeak–Tend Interval

Tpeak–Tend interval (Tp-Te interval) is a well-known marker of ventricular arrhythmogenicity, and an increased Tp-Te interval is an independent risk factor for ventricular arrhythmias (Yagishita et al., 2015). RR and QT intervals were measured from the surface ECG during sinus rhythm. The corrected QT interval was calculated by dividing the QT interval by the square root of the R-R interval. Tp-Te interval was assessed in limb leads with the clearest T-wave at 200 mm/s speed as the average of five consecutive beats. Tp-Te interval was measured from the peak of T wave to the end of the T wave; the peak of the T wave was visually determined, and the end of the T wave was defined as the intersection of the tangent to the slope of the T wave and the isoelectric line (Vaseghi et al., 2013). Tp-Te interval and Tp-Te interval/QTc were compared before and after the DRGS, which were measured 1 min after the S1S2 pacing.

### Hemodynamic Assessment

A 5 French SPR-350 Millar Mikro-Tip pressure transducer catheter (Millar Instruments, Houston, TX) was inserted into the left ventricle *via* the left carotid artery and connected to an MPVS Ultra Pressure Volume Loop System (Millar Instruments, Houston, TX). Left ventricular systolic function was evaluated by the maximum rate of pressure change (dP/dt max).

### Statistical Analysis

All data are expressed as mean ± SE. To compare the data for each stimulation parameter, a paired *t*-test was performed for all measurements. When normality was not seen with Shapiro–Wilk test, the Wilcoxon test was performed. A two-way ANOVA test was performed to compare raw cARI data throughout the experiment. To compare S_max_ between 20 Hz and 1 k Hz, an unpaired *t*-test was performed. For hemodynamic analysis, one-way ANOVA test was performed to compare hemodynamic indexes, and Kruskal-Wallis test was performed only for SBP and dP/dt max at 20 Hz DRGS because the normality was not seen. All figures were created using GraphPad Prism software (version 8, GraphPad Software Inc, San Diego, CA). The sample size was selected based on the previously published work where a large animal model was used to compare the data with and without treatment intervention (Yagishita et al., 2015; Howard-Quijano et al., 2017a,b). A *P*-value ≤ 0.05 was considered statistically significant.

## Results

### Effect of DRGS on Activation Recovery Interval

The MT of DRGS was assessed (0.4 ± 0.05 mA), and the DRGS was applied at 90% of the MT. DRGS alone did not change the ARI with either 20 Hz (baseline: 417 ± 19 ms. DRG30: 415 ± 22 ms, *P* > 0.9) and 1 kHz (baseline: 433 ± 15 ms, DRG30: 431 ± 12 ms, *P* > 0.9).

### Effect of DRGS on Activation Recovery Interval During Ventricular Extrastimulation

**Figure 3:**
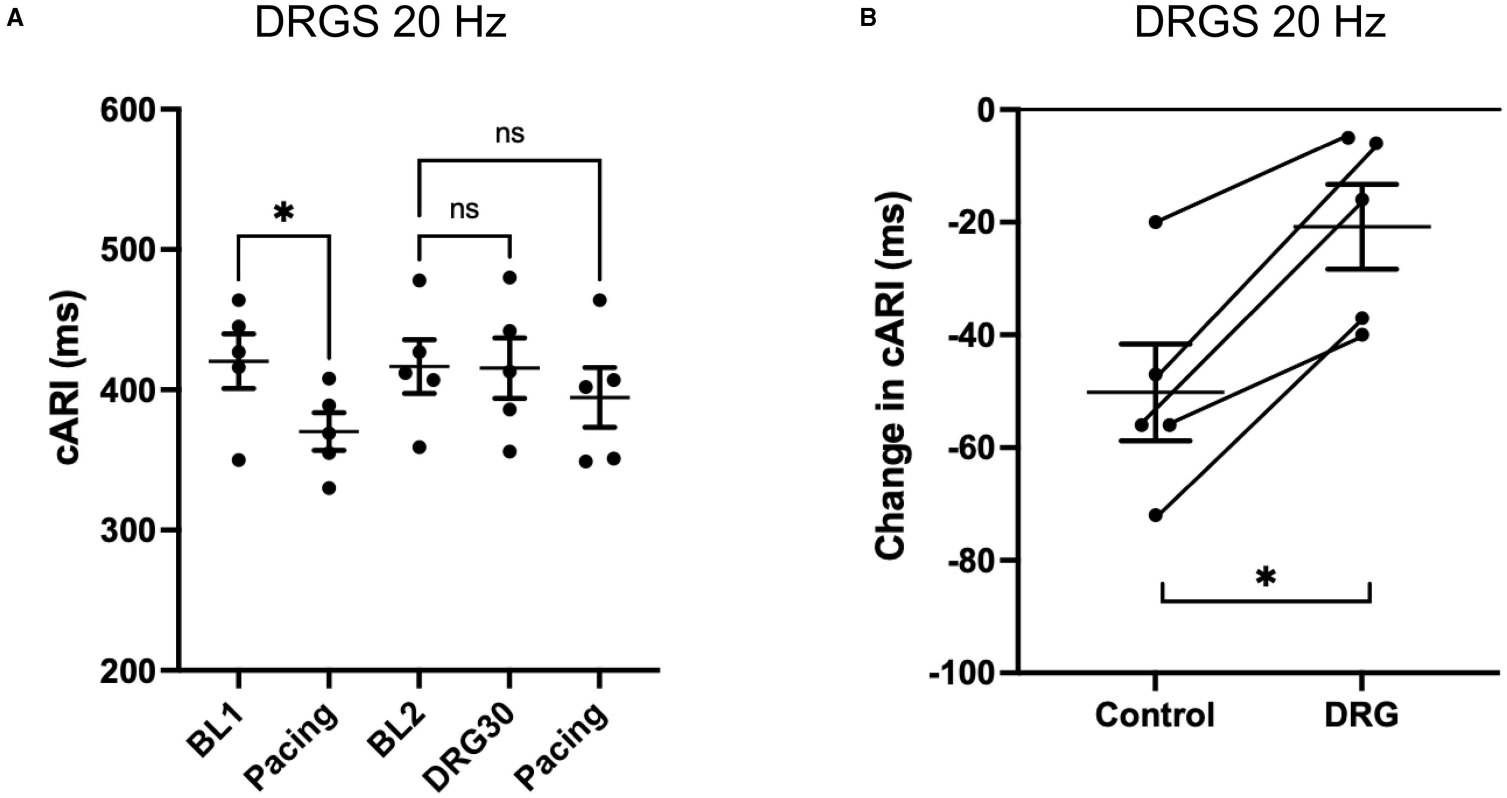
Change in activation recovery interval (ARI) during the experiments with DRG stimulation at 20 Hz. **(A)** Changes in ARI corrected by heart rate (cARI) during experimental protocol. Before DRG stimulation, cARI reduced after the pacing with significant difference (^*^P = 0.414 from BL1), but there was no significant difference after DRG stimulation (*P* = 0.586 from BL2). BL, baseline; S1S2 Pacing, 1 min post-pacing before or after DRG stimulation; DRGS30, 30 min after the initiation of DRG stimulation. **(B)** Reduction of cARI at 1 min post-pacing from baseline or DRG30. DRG stimulation decreased the reduction of cARI with significant difference (^*^P = 0.007). Data are reported as mean ± standard error (SE).

**Figure 4:**
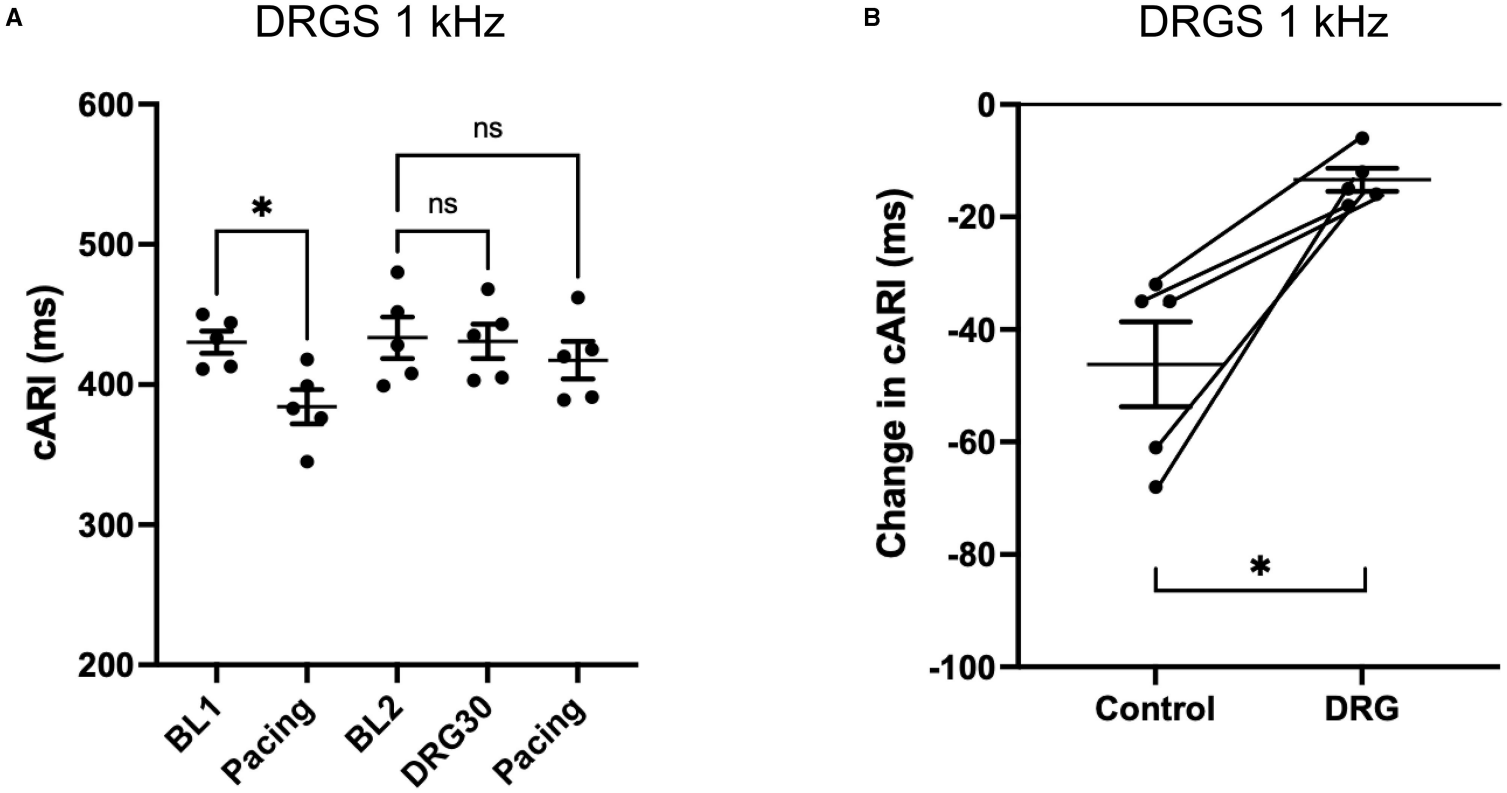
Change in activation recovery interval (ARI) during the experiments with DRG stimulation at 1 kHz. **(A)** Changes in ARI corrected by heart rate (cARI) during the experimental protocol. Before DRG stimulation, cARI reduced after the pacing with significant difference (*P = 0.0378 from BL1), but there was no significant difference after DRG stimulation (*P* = 0.2858 from BL2). BL, baseline; S1S2 Pacing, 1 min post-pacing before or after DRG stimulation; DRG30, 30 min after the initiation of DRG stimulation. **(B)** Reduction of cARI at 1 min post-pacing from baseline or DRG30. DRG stimulation decreased the reduction of cARI with significant difference (*P = 0.013). Data are reported as mean ± standard error (SE).

With the S1S2 ventricular pacing, the reduction in cARI (BL1: 420 ± 19 ms, pacing: 370 ± 13 ms. *P* = 0.041) was mitigated after the 20 Hz DRGS (BL2: 417 ± 19 ms, pacing: 395 ± 21 ms. *P* = 0.586) (Figure 3A). The reduction in cARI caused by the S1S2 ventricular pacing (BL1: 430 ± 8 ms, pacing; 384 ± 12 ms. *P* = 0.038) was also mitigated after 1 kHz DRGS (BL2: 433 ± 15 ms, pacing: 417 ± 13 ms. *P* = 0.286) (Figure 4A).

Dorsal root ganglion stimulation blunted the ventricular pacing-induced cARI reduction with both 20 Hz (Control: −50 ± 9 ms, DRGS: −21 ± 7 ms, *P* = 0.007) (Figure 3B), and 1 kHz (Control: −46 ± 8 ms, DRGS: −13 ± 2 ms, *P* = 0.013) (Figure 4B).

### Change in Electrical Restitution and Ventricular Arrhythmogenicity With DRGS

**Figure 5:**
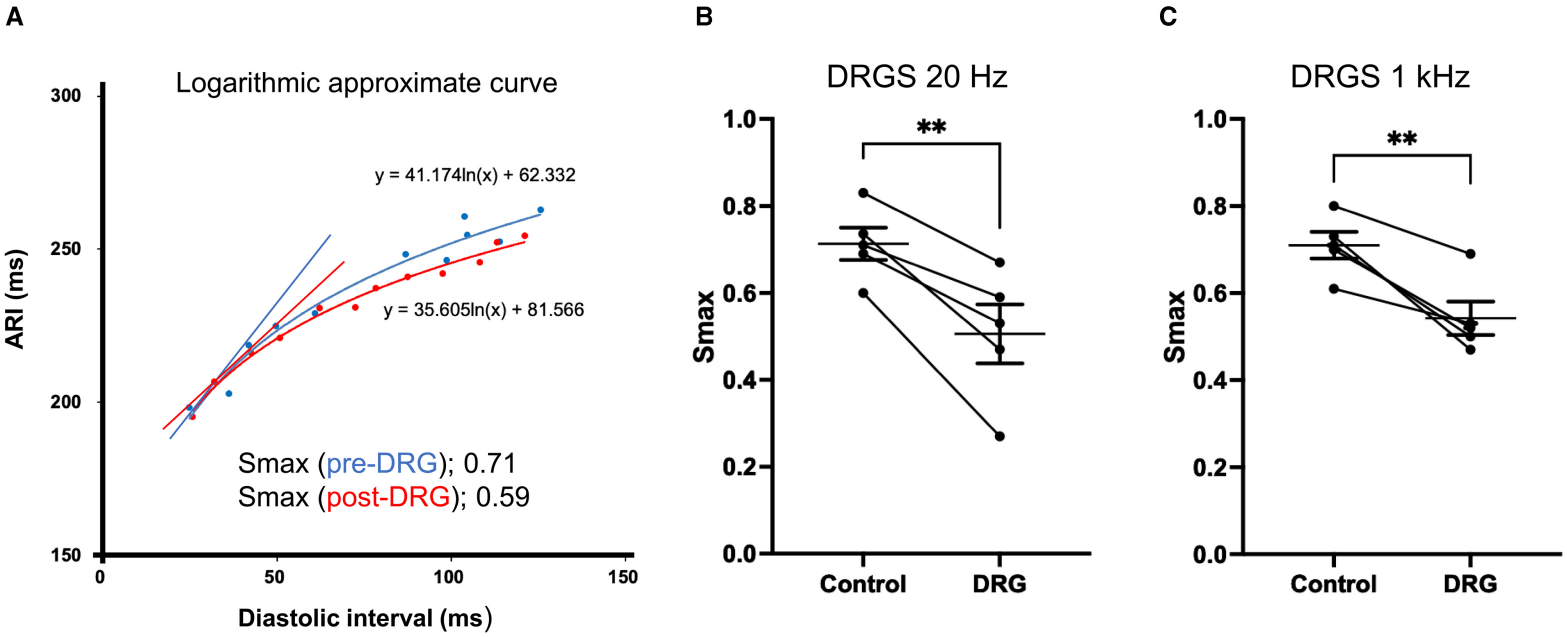
Representative electrical restitution curve and global ventricular maximum slope (S_max_). **(A)** Representative figure of electrical restitution curve showing the decrease in maximum steep slope (Smax) after 30 min of dorsal root ganglion stimulation (DRGS) compared to pre-DRG. Thirty min of DRGS at 20 Hz or 1 kHz decreased S_max_ with significant differences [^**^P = 0.0061 at 20 Hz on the left figure **(B)**, ^**^P = 0.0069 at 1 kHz on the right figure **(C)**]. The data are reported as mean ± standard error (SE).

Electrical restitution curves were derived from recorded ARIs and diastolic intervals (Figure 5A). Smax, the maximal slope, was reduced after DRGS at both frequencies (20 Hz: pre-DRGS: 0.7 ± 0.04, post-DRGS: 0.5 ± 0.07, *P* = 0.0061; 1 kHz: pre-DRGS: 0.7 ± 0.03, post-DRGS 0.5 ± 0.04, *P* = 0.0069) (Figures 5B,C).

There was no statistically significant difference between the magnitude of reduction in Smax between 20 Hz and 1 kHz stimulation (20 Hz: 0.21 ± 0.039, 1 kHz: 0.17 ± 0.031, *P* = 0.49).

### Influence of DRGS on Tp-Te Interval and Risk of Ventricular Arrhythmogenicity

Tpeak-Tend interval and the ratio between Tp-Te interval and corrected QTc were measured from the ECG.

**Figure 6:**
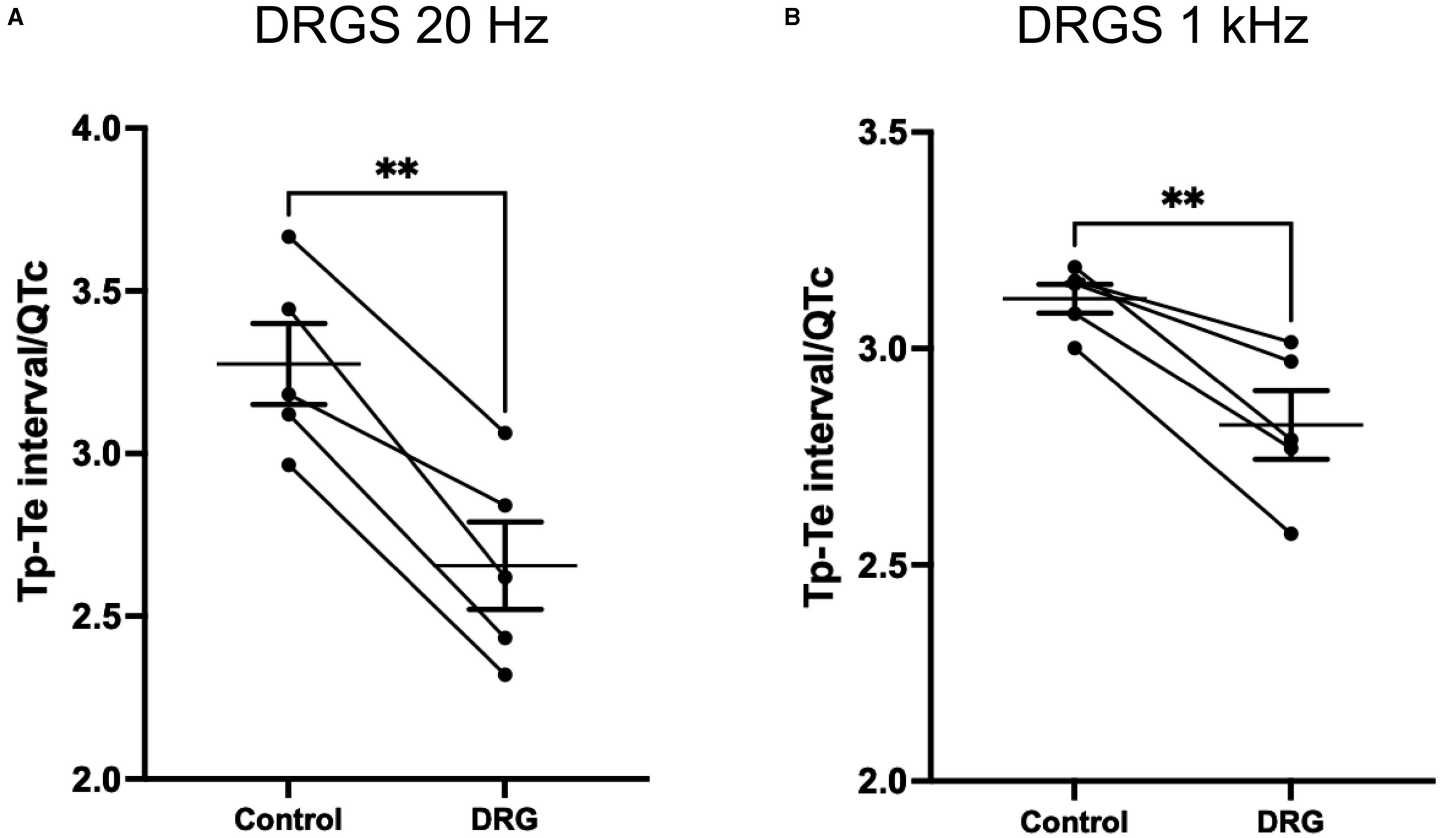
DRG stimulation at both 20 Hz **(A)** and 1 kHz **(B)** decreased Tp-Te nterval/QTc which indicate that DRG stimulation had effect on reducing arrhythmogenesis substrate induced by S1/S2 pacing. Tp-Te measurement was performed at 1 min post S1S2 pacing before and after DRG stimulation. **(A)** **P = 0.014, **(B)** **P = 0.007. Data are reported as mean ± standard error (SE).

At 20 Hz DRGS, Tp-Te interval at pre- and post-DRGS were 46 ± 3.3 ms and 37 ± 3.5 ms (*P* = 0.0053), respectively. Tp-Te interval/QTc showed a significant difference between pre- and post-DRGS (Control; 3.3 ± 0.12, DRGS; 2.7 ± 0.13; *P* = 0.0014) (Figure 6A).

With the 1 kHz DRGS, Tp-Te interval and Tp-Te interval/QTC significantly decreased (Tp-Te interval: pre-DRG; 45 ± 1.3 ms, post-DRGS; 41 ± 1.9 ms. P = 0.021; Tp-Te interval/QTc: Control; 3.1 ± 0.03, DRGS; 2.8 ± 0.08. P = 0.007) (Figure 6B).

### Hemodynamic Response to Dorsal Root Ganglion Stimulation

No significant hemodynamic changes were observed in response to the ventricular extrastimuli before and after DRGS (Supplementary Table 1). DRGS at 20 Hz and 1 kHz did not significantly change the basal heart rate, blood pressure, and contractility (Supplementary Table 1).

## Discussion

In this study, we investigated the efficacy of DRGS on reducing cardiac sympathoexcitation and the indices associated with increased arrhythmogenicity in a porcine model. Augmented adrenergic levels and enhanced sympathetic activity have been shown to increase Smax, shorten ARI, and promote ventricular arrhythmias, suggesting that DRGS might be reducing the sympathetic output (Taggart et al., 2003). We demonstrated that during myocardial stress from S1/S2 pacing, targeting the afferent loop of the cardiac-neural reflex with DRGS (1) reduced cARI shortening, (2) reduced the maximum slope of electrical restitution curve, and (3) decreased Tp-Te interval and Tp-Te interval/QTc.

To our knowledge, this is the first study to report the effectiveness of DRGS on reducing myocardial excitability and risk for ventricular arrhythmogenicity. Single-site DRGS showed an effective afferent neuromodulation in the setting of pain relief (Franken et al., 2018; Piedade et al., 2019); therefore in this study, we chose to stimulate DRG at a single site at the left T2, which would also be a more clinically pragmatic approach. The T2-level selection was based on the fact that multiple cardiac afferent nerve fiber travels to thoracic spinal cords through the C6-T6 DRGs bilaterally, with the largest numbers to the T2-T4 (Hopkins and Armour, 1989), and the fact that cardiac afferents are distributed symmetrically between the left and right sides (Akgul Caglar et al., 2021).

In addition, we studied if both the conventional (20 Hz) and novel higher-frequency (1 kHz) DRG stimulations can modulate the cardio-spinal reflex. Not much has been described with regard to the difference in efficacy of 20 Hz vs. 1 kHz stimulation when used for DRG neuromodulation. A prior report described equally effective pain relief following conventional vs. high-frequency stimulation in an animal model of painful diabetic neuropathy (Koetsier et al., 2020). No difference was observed in our study on any of the outcome measures of cardiac excitability and risk for ventricular arrhythmias. Future research is warranted to demonstrate any benefits of high-frequency stimulation in reduction of paresthesia that is typically associated with conventional stimulation frequencies.

### Dorsal Root Ganglion Stimulation Modulation of Cardiac Sympathetic Activity and Ventricular Excitability

Electrical stimulation of segmental DRG neurons is a new clinical treatment principally introduced for pain syndromes. The mechanism of action of DRG stimulation is through augmentation of T-junction filtering by reducing action potentials from peripheral systems (Chao et al., 2020).

In this study, DRGS prevented the reduction in ARI seen as a result of sympathetic hyperactivity during induced cardiac stress with programmed ventricular extrastimuli (S1S2 pacing). We and others have previously shown that ARI shortening occurs during increases in sympathetic discharge following cardiac stress (Vaseghi et al., 2013; Howard-Quijano et al., 2017a,b; Omura et al., 2021), and neuromodulation can block the cardiospinal neural reflex and prevent exaggerated sympathetic output to the heart (Salavatian et al., 2019). Our results show that DRGS reduced the cardiac sympathetic output *via* controlling the afferent arm of the cardio-neural reflex. In support of our results, a recent study has demonstrated that left-sided DRGS lowers sympathetic nerve activity by significantly reducing the firing frequency of sympathetic nerves (Sverrisdottir et al., 2020).

Mechanisms of ventricular arrhythmias are complex, and the alteration of autonomic nervous activity with augmented sympathetic reflexes or reduced vagal tone strongly influences ventricular arrhythmogenicity (Gilmour, 2001; Antzelevitch, 2007). In the ventricles, increased sympathetic tone may induce abnormal focal activity and reentrant activity, playing a critical role in triggering and maintenance of ventricular arrhythmias (Lai et al., 2019). In our study, we showed that DRGS modified electrophysiological measures of cardiac excitability and reduced ventricular arrhythmogenicity.

Electrical restitution kinetics are performed to predict arrhythmogenicity clinically and experimentally by plotting the relationship between the ARI (a surrogate for APD) and the diastolic intervals (Zhao et al., 2015; Tse et al., 2016). A steep slope of the APD restitution can precipitate ventricular fibrillation by inducing large persistent beat-to-beat APD oscillations that result in a breakup of the electrical wave (Osadchii, 2019). Sympathetic stimulation has been shown to increase electrical restitution and increased the susceptibility of the heart to ventricular fibrillation (Taggart et al., 2003; Ng et al., 2007; Osadchii, 2019).

Tpeak-Tend interval assessments correlate with whole heart dispersion and have a strong link between adrenergic stimulation, proarrhythmia, and the reason behind the strong predictability of Tp-Te interval as a surface ECG marker of sudden cardiac death (Vaseghi et al., 2013). For all animals included in this study, both Smax restitution slope and Tp-Te interval reduced after DRGS, supporting the effectiveness of DRGS for reducing ventricle burdens for arrhythmias.

Importantly, we did not see any significant differences in hemodynamics following DRGS as the heart rate, systolic blood pressure, and dP/dt max remained unaltered. These results are consistent with those with spinal cord stimulation during myocardial ischemia and support the use of DRGS for the selective modulation of ventricular arrhythmogenicity while keeping the hemodynamics stable.

### Limitations

In this study, we have shown that the DRGS reduced ventricular arrhythmogenicity in pigs under anesthesia, which can impact autonomic tone. Since we used alpha-chloralose during the period to test and record cardiac electrophysiology, the impact of DRGS on reducing cardiac excitability during myocardial stress is likely to be preserved. However, it is still possible to see a difference in the DRGS efficacy in awake subjects. For the DRGS parameters, we have assessed the efficacy of two different DRGS parameters (two frequencies with two different pulse widths) based on the previous study (Chapman et al., 2020) as the optimal parameter has not been elucidated yet. Further studies will be necessary to find the optimal DRGS parameters, especially in animal models of cardiac disease. We showed that DRGS decreased cardiac sympathoexcitation and markers of arrhythmogenicity, although the study was not designed to record ventricular arrhythmias themselves. Previous studies have demonstrated that a strong relationship exists between the adrenergic-induced increase in Smax and the shortening of ARI that are associated with ventricular arrhythmias (Taggart et al., 2003).

## Conclusions

Dorsal root ganglion stimulation at both low and high frequencies reduced the ventricular arrhythmogenicity during programmed ventricular extrastimulation in a porcine model. Our result suggests that DRGS, through the modulation of afferent cardiac neural signals at the level of dorsal root ganglia, reduces sympathetic hyperactivity during cardiac stress.

## Data Availability Statement

The raw data supporting the conclusions of this article will be made available by the authors, without undue reservation.

## Ethics Statement

The animal study was reviewed and approved by Institutional Animal Care and Use Committee (IACUC) at the University of Pittsburgh.

## Author Contributions

YK, SS, KH-Q, and AM conceived, designed, analyzed research, and interpreted the results of experiments and drafted the manuscript. YK, TY, and EL conducted experiments. YK analyzed data and prepared figures. All authors reviewed and approved the final version of the manuscript.

## Funding

This study was funded by NIH RO1 HL136836. AM was supported by NIH RO1 HL136836 and NIH R44 DA049630. KH-Q was supported by NIH K08 HL135418.

## Conflict of Interest

The authors declare that the research was conducted in the absence of any commercial or financial relationships that could be construed as a potential conflict of interest.

## Publisher's Note

All claims expressed in this article are solely those of the authors and do not necessarily represent those of their affiliated organizations, or those of the publisher, the editors and the reviewers. Any product that may be evaluated in this article, or claim that may be made by its manufacturer, is not guaranteed or endorsed by the publisher.

## References

[B1] Ajijola O. A. Vaseghi M. Zhou W. Yamakawa K. Benharash P. Hadaya J.. (2013). Functional differences between junctional and extrajunctional adrenergic receptor activation in mammalian ventricle. Am. J. Physiol. Heart Circ. Physiol. 304, H579–588. 10.1152/ajpheart.00754.2012 23241324 PMC3566483

[B2] Akgul Caglar T. Durdu Z. B. Turhan M. U. Gunal M. Y. Aydin M. S. Ozturk G.. (2021). Evaluation of the bilateral cardiac afferent distribution at the spinal and vagal ganglia by retrograde labeling. Brain Res. 1751:147201. 10.1016/j.brainres.2020.147201 33171152

[B3] Antzelevitch C. (2007). Role of spatial dispersion of repolarization in inherited and acquired sudden cardiac death syndromes. Am. J. Physiol. Heart Circ. Physiol. 293, H2024–2038. 10.1152/ajpheart.00355.2007 17586620 PMC2085107

[B4] Barman S. M. Yates B. J. (2017). Deciphering the neural control of sympathetic nerve activity: status report and directions for future research. Front. Neurosci. 11:730. 10.3389/fnins.2017.00730 29311801 PMC5743742

[B5] Batul S. A. Olshansky B. Fisher J. D. Gopinathannair R. (2017). Recent advances in the management of ventricular tachyarrhythmias. F1000Res 6:1027. 10.12688/f1000research.11202.1 28721212 PMC5497814

[B6] Chao D. Zhang Z. Mecca C. M. Hogan Q. H. Pan B. (2020). Analgesic dorsal root ganglionic field stimulation blocks conduction of afferent impulse trains selectively in nociceptive sensory afferents. Pain 161, 2872–2886. 10.1097/j.pain.0000000000001982 32658148 PMC7669706

[B7] Chapman K. B. Yousef T. A. Vissers K. C. Van Helmond N. and M. D. S.-H. (2020). Very low frequencies maintain pain relief from dorsal root ganglion stimulation: an evaluation of dorsal root ganglion neurostimulation frequency tapering. Neuromodulation 24, 746–752. 10.1111/ner.13322 33227827

[B8] Chinushi M. Tagawa M. Kasai H. Washizuka T. Abe A. Furushima H.. (2001). Correlation between the effective refractory period and activation-recovery interval calculated from the intracardiac unipolar electrogram of humans with and without dl-Sotalol treatment. Jpn. Circ. J. 65, 702–706. 10.1253/jcj.65.702 11502045

[B9] Dale E. A. Kipke J. Kubo Y. Sunshine M. D. Castro P. A. Ardell J. L.. (2020). Spinal cord neural network interactions: implications for sympathetic control of the porcine heart. Am. J. Physiol. Heart Circ. Physiol. 318, H830–H839. 10.1152/ajpheart.00635.2019 32108524 PMC7191487

[B10] Deer T. R. Pope J. E. Lamer T. J. Grider J. S. Provenzano D. Lubenow T. R.. (2019). The neuromodulation appropriateness consensus committee on best practices for dorsal root ganglion stimulation. Neuromodulation 22, 1–35. 10.1111/ner.12845 30246899

[B11] Dusi V. Zhu C. Ajijola O. A. (2019). Neuromodulation approaches for cardiac arrhythmias: recent advances. Curr. Cardiol. Rep. 21:32. 10.1007/s11886-019-1120-1 30887264 PMC7105505

[B12] Franken G. Debets J. Joosten E. a. J. (2018). Dorsal root ganglion stimulation in experimental painful diabetic peripheral neuropathy: burst vs. conventional stimulation paradigm. Neuromodulation 22, 943–950. 10.1111/ner.12908 30570187 PMC7027839

[B13] Fukuda K. Kanazawa H. Aizawa Y. Ardell J. L. Shivkumar K. (2015). Cardiac innervation and sudden cardiac death. Circ. Res. 116, 2005–2019. 10.1161/CIRCRESAHA.116.304679 26044253 PMC4465108

[B14] Gilmour R. F. (2001). Life out of balance: the sympathetic nervous system and cardiac arrhythmias. Cardiovasc. Res. 51, 625–626. 10.1016/S0008-6363(01)00402-3 11530093

[B15] Herring N. Kalla M. Paterson D. J. (2019). The autonomic nervous system and cardiac arrhythmias: current concepts and emerging therapies. Nat. Rev. Cardiol. 16, 707–726. 10.1038/s41569-019-0221-2 31197232

[B16] Hopkins D. A. Armour J. A. (1989). Ganglionic distribution of afferent neurons innervating the canine heart and cardiopulmonary nerves. J. Auton. Nerv. Syst. 26, 213–222. 10.1016/0165-1838(89)90170-7 2754177

[B17] Howard-Quijano K. Takamiya T. Dale E. A. Kipke J. Kubo Y. Grogan T.. (2017a). Spinal cord stimulation reduces ventricular arrhythmias during acute ischemia by attenuation of regional myocardial excitability. Am. J. Physiol. Heart Circ. Physiol. 313, H421–H431. 10.1152/ajpheart.00129.2017 28576833 PMC5582923

[B18] Howard-Quijano K. Takamiya T. Dale E. A. Yamakawa K. Zhou W. Buckley U.. (2017b). Effect of thoracic epidural anesthesia on ventricular excitability in a porcine model. Anesthesiology 126, 1096–1106. 10.1097/ALN.0000000000001613 28358748

[B19] Koetsier E. Franken G. Debets J. Van Kuijk S. M. J. Linderoth B. Joosten E. A.. (2020). Dorsal root ganglion stimulation in experimental painful diabetic polyneuropathy: delayed wash-out of pain relief after low-frequency (1Hz) stimulation. Neuromodulation 23, 177–184. 10.1111/ner.13048 31524325

[B20] Lai Y. Yu L. Jiang H. (2019). Autonomic neuromodulation for preventing and treating ventricular arrhythmias. Front. Physiol. 10:200. 10.3389/fphys.2019.00200 30914967 PMC6421499

[B21] Liem L. Russo M. Huygen F. J. P. M. Van Buyten J.-P. Smet I. Verrills P.. (2015). One-year outcomes of spinal cord stimulation of the dorsal root ganglion in the treatment of chronic neuropathic pain. Neuromodulation 18, 41–49. 10.1111/ner.12228 25145467

[B22] Lopshire J. C. Zhou X. Dusa C. Ueyama T. Rosenberger J. Courtney N.. (2009). Spinal cord stimulation improves ventricular function and reduces ventricular arrhythmias in a canine postinfarction heart failure model. Circulation 120, 286–294. 10.1161/CIRCULATIONAHA.108.812412 19597055

[B23] Millar C. K. Kralios F. A. Lux R. L. (1985). Correlation between refractory periods and activation-recovery intervals from electrograms: effects of rate and adrenergic interventions. Circulation 72, 1372–1379. 10.1161/01.CIR.72.6.1372 4064279

[B24] Ng G. A. Brack K. E. Patel V. H. Coote J. H. (2007). Autonomic modulation of electrical restitution, alternans and ventricular fibrillation initiation in the isolated heart. Cardiovasc. Res. 73, 750–760. 10.1016/j.cardiores.2006.12.001 17217937

[B25] Omura Y. Kipke J. P. Salavatian S. Afyouni A. S. Wooten C. Herkenham R. F.. (2021). Spinal anesthesia reduces myocardial ischemia-triggered ventricular arrhythmias by suppressing spinal cord neuronal network interactions in pigs. Anesthesiology 134, 405–420. 10.1097/ALN.0000000000003662 33411921 PMC7878394

[B26] Osadchii O. E. (2012). Effects of ventricular pacing protocol on electrical restitution assessments in guinea-pig heart. Exp. Physiol. 97, 807–821. 10.1113/expphysiol.2012.065219 22447974

[B27] Osadchii O. E. (2019). Effects of antiarrhythmics on the electrical restitution in perfused guinea-pig heart are critically determined by the applied cardiac pacing protocol. Exp. Physiol. 104, 490–504. 10.1113/EP087531 30758086

[B28] Pak H. N. Hong S. J. Hwang G. S. Lee H. S. Park S. W. Ahn J. C.. (2004). Spatial dispersion of action potential duration restitution kinetics is associated with induction of ventricular tachycardia/fibrillation in humans. J. Cardiovasc. Electrophysiol. 15, 1357–1363. 10.1046/j.1540-8167.2004.03569.x 15610278

[B29] Piedade G. S. Vesper J. Chatzikalfas A. Slotty P. J. (2019). Cervical and high-thoracic dorsal root ganglion stimulation in chronic neuropathic pain. Neuromodulation 22, 951–955. 10.1111/ner.12916 30620789

[B30] Proietti R. Joza J. Essebag V. (2016). Therapy for ventricular arrhythmias in structural heart disease: a multifaceted challenge. J. Physiol. 594, 2431–2443. 10.1113/JP270534 26621333 PMC4850198

[B31] Rubart M. Zipes D. P. (2005). Mechanisms of sudden cardiac death. J. Clin. Invest. 115, 2305–2315. 10.1172/JCI26381 16138184 PMC1193893

[B32] Salavatian S. Ardell S. M. Hammer M. Gibbons D. Armour J. A. Ardell J. L. (2019). Thoracic spinal cord neuromodulation obtunds dorsal root ganglion afferent neuronal transduction of the ischemic ventricle. Am. J. Physiol. Heart Circ. Physiol. 317, H1134–H1141. 10.1152/ajpheart.00257.2019 31538809 PMC6879916

[B33] Shen M. J. Zipes D. P. (2014). Role of the autonomic nervous system in modulating cardiac arrhythmias. Circ. Res. 114, 1004–1021. 10.1161/CIRCRESAHA.113.302549 24625726

[B34] Southerland E. M. Milhorn D. M. Foreman R. D. Linderoth B. Dejongste M. J. Armour J. A.. (2007). Preemptive, but not reactive, spinal cord stimulation mitigates transient ischemia-induced myocardial infarction via cardiac adrenergic neurons. Am. J. Physiol. Heart Circ. Physiol. 292, H311–317. 10.1152/ajpheart.00087.2006 16920800

[B35] Sverrisdottir Y. B. Martin S. C. Hadjipavlou G. Kent A. R. Paterson D. J. Fitzgerald J. J.. (2020). Human dorsal root ganglion stimulation reduces sympathetic outflow and long-term blood pressure. JACC Basic Transl. Sci. 5, 973–985. 10.1016/j.jacbts.2020.07.010 33145461 PMC7591825

[B36] Taggart P. Sutton P. Chalabi Z. Boyett M. R. Simon R. Elliott D.. (2003). Effect of adrenergic stimulation on action potential duration restitution in humans. Circulation 107, 285–289. 10.1161/01.CIR.0000044941.13346.74 12538429

[B37] Tse G. Wong S. T. Tse V. Yeo J. M. (2016). Restitution analysis of alternans using dynamic pacing and its comparison with S1S2 restitution in heptanol-treated, hypokalaemic Langendorff-perfused mouse hearts. Biomed. Rep. 4, 673–680. 10.3892/br.2016.659 27284405 PMC4887808

[B38] Vaseghi M. Yamakawa K. Sinha A. So E. L. Zhou W. Ajijola O. A.. (2013). Modulation of regional dispersion of repolarization and T-peak to T-end interval by the right and left stellate ganglia. Am. J. Physiol. Heart Circ. Physiol. 305, H1020–1030. 10.1152/ajpheart.00056.2013 23893168 PMC3798747

[B39] Yagishita D. Chui R. W. Yamakawa K. Rajendran P. S. Ajijola O. A. Nakamura K.. (2015). Sympathetic nerve stimulation, not circulating norepinephrine, modulates T-peak to T-end interval by increasing global dispersion of repolarization. Circ. Arrhythm. Electrophysiol. 8, 174–185. 10.1161/CIRCEP.114.002195 25532528 PMC4405126

[B40] Yu G. Segel I. Zhang Z. Hogan Q. H. Pan B. (2020). Dorsal root ganglion stimulation alleviates pain-related behaviors in rats with nerve injury and osteoarthritis. Anesthesiology 133, 408–425. 10.1097/ALN.0000000000003348 32433276 PMC8195267

[B41] Zhao D. Liu B. Wei Y. Tang K. Yu X. Xu Y. (2015). The roles of pacing interval and pacing strength in ventricular fibrillation induced by rapid pacing with 1:1 capture. Arch. Med. Sci. 11, 1111–1118. 10.5114/aoms.2015.54868 26528357 PMC4624755

[B42] Zucker I. H. (2006). Novel mechanisms of sympathetic regulation in chronic heart failure. Hypertension 48, 1005–1011. 10.1161/01.HYP.0000246614.47231.25 17015773

