# Supplementary material for: Neuromodulation With Thoracic Dorsal Root Ganglion Stimulation Reduces Ventricular Arrhythmogenicity
Source: Front Physiol. 2021 Oct 7;12:713717. doi: 10.3389/fphys.2021.713717 (PMC8528951; doi:10.3389/fphys.2021.713717)
Supplement: Supplementary file 1 [file Table_1.pdf]

Table 1. Hemodynamic changes with 20 Hz and 1 kHz DRGS

		Baseline-1	S1S2-1	Baseline-2	DRGS-30	S1S2-2
**HR** (bpm)	**20 Hz**	87 ± 9	87 ± 9	89 ± 9	90 ± 10	90 ± 9
	**1 kHz**	79 ± 8	80 ± 7	79 ± 9	80 ± 9	83 ± 8
**SBP** (mmHg)	**20 Hz**	129 ± 6	132 ± 8	128 ± 7	130 ± 7	135 ± 9
	**1 kHz**	125 ± 9	134 ± 6	135 ± 7	128 ± 9	124 ± 15
**dP/dt max** (mmHg/s)	**20 Hz**	1870 ± 75	1766 ± 116	1797 ± 55	1773 ± 98	1816 ± 88
	**1 kHz**	1959 ± 314	1723 ± 246	1753 ± 203	1675 ± 274	2028 ± 575

Dorsal root ganglion stimulation (DRGS) both at 20 Hz and 1 kHz showed no significant differences on the hemodynamic indexes measured by HR, SBP, and dP/dt max (All $P > 0.9$). Data is presented as mean ± standard error (SE). HR (beat/min); Heart Rate, SBP (mmHg); Systolic blood pressure, dP/dt max (mmHg/s); the maximum rate of pressure change. n; animal numbers. S1S2 Pacing-1; 1 minute after the first pacing without DRGS. DRGS-30; 30 minutes after DRGS. S1S2 Pacing-2; 1 minute after the second pacing with DRGS.
